# Peer pressure and adolescent mobile social media addiction: Moderation analysis of self-esteem and self-concept clarity

**DOI:** 10.3389/fpubh.2023.1115661

**Published:** 2023-04-11

**Authors:** Xiaopan Xu, Wanqu Han, Qingqi Liu

**Affiliations:** ^1^Institute for Public Policy and Social Management Innovation, College of Political Science and Public Administration, Henan Normal University, Xinxiang, China; ^2^School of Sociology, Central China Normal University, Wuhan, China; ^3^College of Education for the Future, Beijing Normal University, Zhuhai, China; ^4^School of Education, Guangzhou University, Guangzhou, China

**Keywords:** peer pressure, mobile social media addiction, self-esteem, self-concept clarity, adolescents

## Abstract

**Background:**

Social media addiction has increasingly been a critical social problem. We explored the association between peer pressure on mobile phone use and adolescent mobile social media addiction and tested whether self-esteem and self-concept clarity could buffer the effect of peer pressure.

**Methods:**

830 adolescents (*M*_age_ = 14.480, SD_age_ = 1.789) participated in our anonymous cross-sectional questionnaire study.

**Results:**

The results showed that peer pressure significantly predicted adolescent mobile social media addiction. Self-esteem moderated the effect of peer pressure on mobile social media addiction in that peer pressure had a weaker effect for adolescents with higher self-esteem. Self-concept clarity moderated the effect of peer pressure on mobile social media addiction in that peer pressure had a weaker effect for adolescents with higher self-esteem. The two moderators also interact in that the moderation of self-esteem was stronger for adolescents with higher self-concept clarity and the moderation of self-concept clarity for adolescents with higher self-esteem.

**Conclusion:**

The results highlight the critical role of self-esteem and self-concept clarity in buffering the impact of peer pressure on mobile social media addiction. The findings promote a better understanding of how to buffer the undesirable effect of peer pressure and reduce the risk of mobile social media addiction among adolescents.

## 1. Introduction

Mobile phone addiction has increasingly become one of the most serious problem behaviors among adolescents (1). Social function is a core function of mobile phones. The function of mobile social is even more important than mobile games because the desire for mobile games only exists in some people, but there is a universal demand for mobile social networking services. Mobile social media addiction is considered the core sub-type of mobile addiction, and it has attracted attention from more and more researchers (2, 3). The effects of social media addiction on individuals’ academic, emotional, and mental health have been examined in some studies. For instance, social media addiction had an undesirable effect on adolescent and young adults’ academic performance (4). Social network site addiction positively predicted loneliness and unmet interpersonal needs, which, in turn, increased the risk of depression (5). Due to the personalized functions and convenient operations of mobile phones, mobile phone social media addiction may have a higher incidence in adolescents. Therefore, exploring the risk factors and buffering mechanisms of mobile social media addiction will greatly help understand the causes and intervention measures of adolescents’ mobile social media addiction.

Peer pressure may be an essential predictor of adolescent mobile social media addiction. Peer pressure refers to the pressure that individuals feel when they are directly or indirectly asked to think and act according to the rules or requirements of their peers (6, 7). Since peer pressure can bring about both positive and negative impacts, it should be analyzed specifically regarding the different stress contents (8, 9). More and more researchers are now exploring peer pressure based on specific stressors (such as peer pressure on Internet use) (2, 7) so as to better describe the impacts of peer pressure on adolescents’ physical and mental development. Peer pressure on Internet use/mobile phone use is the pressure individuals feel when they are directly or indirectly promoted to use the Internet/mobile phones to maintain and develop peer relationships. Research has found that peer pressure on Internet use is one of the key risk factors for adolescents’ Internet addiction (7). Nowadays, mobile phones have become the dominant tool for adolescents to communicate online. Peer pressure on mobile phone use has become the main type of peer pressure adolescents experience daily. According to the theory of peer norm influence (10), when a certain behavior becomes one of the normative behaviors of peer groups, it will exert pressure on adolescents and drive them to carry out such behaviors. Only in this way can adolescents conform to peer norms, maintain peer relationships, and meet the needs of belonging. Peer pressure on mobile phone use will make adolescents use mobile phones frequently under pressure. Thus, we hypothesized that peer pressure on mobile phone use would directly and positively predict adolescents’ mobile social media addiction (Hypothesis 1).

In addition, according to the individual-environment interaction model (11, 12), environmental and individual factors do not exert effects independently, and they often jointly affect people’s physical and mental development. Therefore, although peer pressure may have a direct impact on adolescent mobile social media addiction, the impact of peer pressure on mobile phone use may vary with individuals’ personality traits. Self-esteem and self-concept clarity, as the core components of self-concept, are two personality traits that can play positive roles in mental health (13, 14). Self-esteem reflects whether an individual has a positive self-concept (15), and self-concept clarity reflects whether an individual has a clear self-concept (16). Research has documented that positive self-esteem and clear self-concept clarity can help adolescents improve academic performance (17, 18), weaken negative emotions (19, 20), and reduce problem behaviors such as aggressive behaviors (21, 22) and addictive behaviors (23, 24). The effects of self-esteem and self-concept clarity on mobile phone addiction have also been verified by previous studies (25, 26).

Moreover, self-esteem and self-concept clarity may act as protective factors to alleviate the impact of negative environmental factors on individuals. According to sociometer theory (27), self-esteem can help individuals regulate social evaluation process. Social self-evaluations of individuals with low self-esteem are significantly more negative, and they are more likely to attribute stress or rejection to internal reasons. On the contrary, individuals with high self-esteem have significantly more positive social self-evaluations, and they tend to attribute stress or exclusion to external causes (28). Research has demonstrated that self-concept clarity can also significantly influence the self-evaluation process (29), and individuals with high self-concept clarity are more inclined to act relying on self-information (30). Thus, self-esteem and self-concept may help adolescents alleviate the risk effects of negative interpersonal factors.

Previous studies have also confirmed the protective effects of self-esteem and self-concept clarity in buffering negative interpersonal factors. For instance, self-esteem moderated the effect of relational victimization on children’s internal problem behaviors, with the effect being stronger for children with lower levels of self-esteem (31). Individuals with lower levels of self-esteem had more risky health behaviors on high-rejection days (32). In addition, research has found that the effect of attachment anxiety on Facebook fatigue through Facebook anxiety was significant at low self-concept clarity but not at high self-concept clarity (33). Adolescents are in the key stage of self-concept development. High self-esteem and self-concept clarity may enable individuals to think and act according to their inner self-concepts. They could thus be less affected by negative peer relationships. In other words, positive and clear self-concept may help adolescents weaken the negative influence of peer pressure and reduce the risk of addiction caused by peer pressure. Thus, we put forward the following two hypotheses:

*Hypothesis 1*: Self-esteem would moderate the association between peer pressure and mobile social media addiction in that peer pressure had a weaker effect for individuals with high self-esteem than for those with low self-esteem.

*Hypothesis 2*: Self-concept clarity would moderate the association between peer pressure and mobile social media addiction in that peer pressure had a weaker effect for individuals with high self-concept clarity than for those with low self-concept clarity.

Although peer pressure has been proven to have a significant predictive effect on adolescents’ Internet addiction (7), the effect of peer pressure on mobile phone addiction is still rarely explored, and whether some positive factors can buffer between peer pressure and adolescent mobile phone social media addiction is also lack of research. Although self-esteem and self-concept clarity may alleviate the negative effects of risk factors on individual mental health and problematic behaviors (31–33), whether they can still play a buffering role in weakening the impact of peer pressure on adolescents’ mobile phone addiction has not been examined by research.

In conclusion, the present study explored the association between peer pressure on mobile phone use and mobile social media addiction and further tested whether self-esteem and self-concept clarity could moderate the effect of peer pressure. In addition to the two simple moderating effects, the present study also examined whether there were significant gender differences in the two moderating effects and whether the two moderators interacted. The results can better reveal the complex protective roles of self-teem and self-concept clarity.

## 2. Materials and methods

### 2.1. Participants and procedure

We invited 896 adolescents from two high schools in South China. They participated in our anonymous survey in the classrooms. Thirty-eight students did not complete all the psychological scales. Twenty-eight students provided invalid responses. Data from 830 adolescents were analyzed to test our hypotheses. Three hundred and seventy-seven adolescents were boys (45.40%), and 453 adolescents (54.60%) were girls. These participants’ age range from 11 to 18 years old (*M*_age_ = 14.480, SD_age_ = 1.789). The detailed demographic data analysis of participants is presented in Table 1.

**Table 1:** Characteristics of study sample.

Characteristics		Number (percentage)
Age	11 years old	10 (1.2%)
	12 years old	152 (18.3%)
	13 years old	103 (12.4%)
	14 years old	160 (19.3%)
	15 years old	133 (16.0%)
	16 years old	130 (15.7%)
	17 years old	126 (15.2%)
	18 years old	16 (1.9%)
Sex	Male	377 (45.4%)
	Female	453 (54.6%)
Residence	Urban	696 (83.9%)
	Rural	134 (16.1%)
Education	Middle school	498 (60.0%)
	High school	332 (40.0%)
Family income	Less than 10,000 RMB	3 (0.4%)
	10,000–29,999 RMB	35 (4.2%)
	30,000–79,999 RMB	123 (14.8)
	80,000–149,999 RMB	226 (27.2%)
	149,999–299,999 RMB	403 (48.6%)
	299,999–999,999 RMB	27 (3.3%)
	1,000,000 RMB or above	13 (1.6%)
Father’s Education	No formal education or illiterate	11 (1.3%)
	Did not finish elementary school	28 (3.4%)
	Elementary school	61 (7.3%)
	Middle school	293 (35.3%)
	High school	345 (41.6%)
	University	92 (11.1%)
Mother’s Education	No formal education or illiterate	19 (2.3%)
	Did not finish elementary school	31 (3.7%)
	Elementary school	75 (9.0%)
	Middle school	335 (40.4%)
	High school	304 (36.6%)
	University	66 (8.0%)
Family supervision	Lives alone	29 (3.5%)
	With family	721 (86.9%)
	With relatives	80 (9.6%)

### 2.2. Measurements

#### 2.2.1. Peer pressure on mobile phone use

The Peer Pressure on Mobile Phone Use Scale (2), adapted from the Chinese version of the Peer Pressure Internet Use Scale (34), was used. It consists of five items. A sample item is “I overuse my mobile phone because of the request of my friends.” These items are rated on a five-point scale (1 = never, 5 = always). High scores represent great peer pressure on mobile phone use. Cronbach’s α for the Peer Pressure on Mobile Phone Use Scale was 0.933.

#### 2.2.2. Mobile social media addiction

The Mobile Social Addiction subscale of the Mobile Phone Addiction Type Scale (MPATS) was used (3). This scale includes six items. A sample item is “I cannot stand not looking at the social apps on my phone for a period of time.” These items are rated on a five-point scale (1 = never, 5 = always). High scores reflect high mobile social media addiction. MPATS has shown good reliability and validity in Chinese adolescents. Cronbach’s α for the Mobile Social Addiction subscale was 0.924.

#### 2.2.3. Self-esteem

The Chinese version (35) of the Rosenberg Self-esteem Scale (36) was adopted. The original measure consists of 10 items rated on a four-point scale (1 = strongly disagree, 4 = strongly agree). Due to the potential cultural differences, one item (“I wish I could have more respect for myself”) was deleted (37). High scores indicate high levels of self-esteem. Cronbach’s α for the self-esteem scale was 0.866.

#### 2.2.4. Self-concept clarity

The Chinese version (38) of the Self-concept Clarity Scale (39) was adopted. There are 12 items rated on a five-point scale (1 = strongly disagree, 5 = strongly agree). A sample item is “I spend a lot of time wondering about what a kind of person I really am.” High scores indicate high self-concept clarity. Cronbach’s α for the self-concept clarity scale was 0.856.

### 2.3. Main statistical analyses

The Pearson correlation analysis was conducted before the model test. We adopted the PROCESS macro for SPSS (40) to analyze the moderating effect of self-esteem, self-concept clarity, and the interaction of the two moderators. The PROCESS has been widely used in previous studies to reveal the mediating effects, moderating effects, and the combined complex effects of mediators and moderators (41). Age (42), gender (43), and daily use time (44, 45) were included in the regression model to control their potential effects.

## 3. Results

### 3.1. Preliminary analysis

Table 2 presents the results of the Pearson correlation analysis. Peer pressure was positively correlated with mobile social media addiction and negatively correlated with self-esteem and self-concept clarity. Self-esteem was positively correlated with self-concept clarity. Both self-esteem and self-concept clarity were negatively correlated with mobile social media addiction.

**Table 2:** Descriptive statistics and intercorrelations between variables.

Variables	*M*	SD	1	2	3	4
1. Peer pressure	2.530	1.159	—			
2. self-esteem	2.598	0.584	−0.162^***^	—		
3. Self-concept clarity	2.814	0.713	−0.218^***^	0.466^***^	—	
4. Mobile social media addiction	2.571	1.266	0.268^***^	−0.259^***^	−0.225^***^	—

*N* = 830. ^***^p < 0.001.

### 3.2. Testing for the moderating roles of self-esteem and self-concept clarity

We conducted two moderation analyses to test the moderating roles of self-esteem and self-concept clarity. The three-way interaction analysis was also performed to examine whether the two moderators interacted with each other.

**Figure 1:**
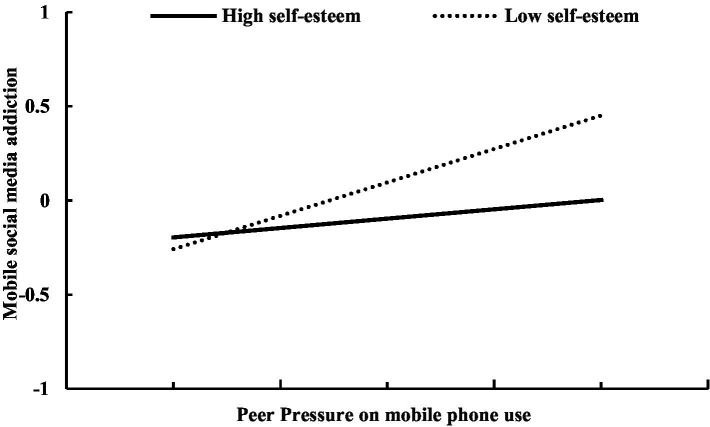
The relationship between peer pressure on mobile phone use and mobile social media addiction at two levels of self-esteem: (1) low self-esteem (1 SD below the mean) and (2) high self-esteem (1 SD above the mean).

Table 3 presents the moderation analysis of self-esteem. After controlling for age, gender, and daily mobile phone use time, peer pressure positively predicted mobile social media addiction (*β* = 0.196, *p* < 0.001), self-esteem negatively predicted mobile social media addiction (*β* = −0.165, *p* < 0.001), and the interaction of peer pressure and self-esteem showed a significant effect on mobile social media addiction (*β* = −0.189, *p* < 0.001). The association between peer pressure and mobile social media addiction was strong in adolescents with low self-esteem (*β* = 0.385, *p* < 0.001) but was not significant for adolescents with high self-esteem (*β* = 0.007, *p* = 0.887). The effect of peer pressure on mobile social media addiction at different levels of self-esteem is depicted in Figure 1.

**Table 3:** Moderation analysis of the role of self-esteem.

Moderation model for predicting mobile social media addiction	*β*	SE	*t*	*p*
Constant	−0.413^***^	0.097	−4.243	< 0.001
Age	−0.088^**^	0.031	−2.863	< 0.01
Gender	0.248^***^	0.062	3.993	< 0.001
Daily mobile phone use time	0.141^***^	0.035	4.000	< 0.001
Peer pressure	0.196^***^	0.033	5.890	< 0.001
Self-esteem	−0.165^***^	0.039	−4.259	< 0.001
Peer pressure × Self-esteem	−0.189^***^	0.030	−6.244	< 0.001
Conditional direct effect analysis at values of the moderator (self-esteem)	*β*	Boot SE	BootLLCI	BootULCI
M − 1 SD (1.55)	0.385^***^	0.043	0.301	0.469
M (2.37)	0.196^***^	0.033	0.131	0.261
M + 1 SD (3.19)	0.007	0.047	−0.086	0.099

*N* = 830. Bootstrap sample size = 5,000. LL, low limit; CI, confidence interval, UL, upper limit. ^**^p < 0.01. ^***^p < 0.001.

**Figure 2:**
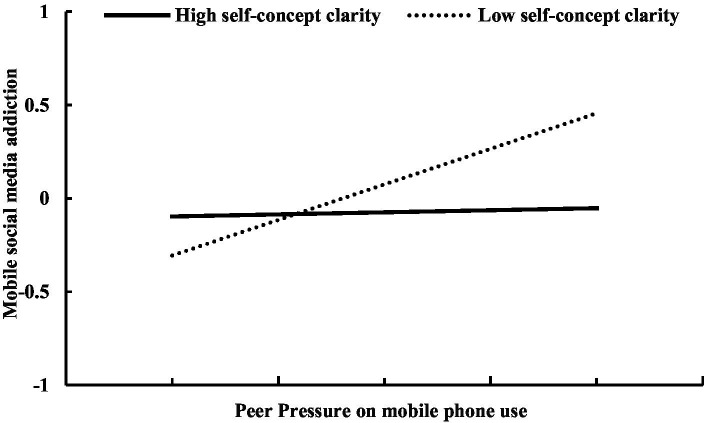
The relationship between peer pressure on mobile phone use and mobile social media addiction at two levels of self-concept clarity: (1) low self-concept clarity (1 SD below the mean) and (2) high self-concept clarity (1 SD above the mean).

Table 4 presents the moderation analysis of self-concept clarity. After controlling for age, gender, and daily mobile phone use time, peer pressure on mobile phone use positively predicted mobile social media addiction (*β* = 0.174, *p* < 0.001), self-concept clarity negatively predicted mobile social media addiction (*β* = −0.105, *p* < 0.01), and the interaction of peer pressure and self-concept clarity showed a significant effect on mobile social media addiction (*β* = −0.217, *p* < 0.001). The association between peer pressure and mobile social media addiction was strong in adolescents with low self-concept clarity (*β* = 0.396, *p* < 0.001) but was not significant for adolescents with high self-concept clarity (*β* = −0.043, *p* = −0.830). The effect of peer pressure on mobile social media addiction at different levels of self-concept clarity is depicted in Figure 2.

**Table 4:** Moderation analysis of the role of self-concept clarity.

Moderation model for predicting mobile social media addiction	*β*	SE	*t*	*p*
Constant	−0.359^***^	0.100	−3.602	< 0.001
Age	−0.089^**^	0.031	−2.851	< 0.01
Gender	0.202^**^	0.063	3.191	< 0.01
Daily mobile phone use time	0.147^***^	0.037	3.975	< 0.001
Peer pressure	0.174^***^	0.035	5.035	< 0.001
Self-concept clarity	−0.105^**^	0.037	−2.873	< 0.01
Peer pressure × Self-concept clarity	−0.217^***^	0.031	−6.934	< 0.001
Conditional direct effect analysis at values of the moderator (Self-concept clarity)	*β*	Boot SE	BootLLCI	BootULCI
M − 1 SD (1.55)	0.396^***^	0.041	0.310	0.472
M (2.37)	0.714^***^	0.035	0.106	0.242
M + 1 SD (3.19)	−0.043	0.051	−0.144	0.058

*N* = 830. Bootstrap sample size = 5,000. LL, low limit; CI, confidence interval; UL, upper limit. ^**^p < 0.01. ^***^p < 0.001.

After controlling for age, gender, and daily mobile phone use time, there was significant three-way interaction between peer pressure, self-esteem, and self-concept clarity (*β* = −0.113, *p* < 0.001). Table 5 presents the results of the conditional moderating effects of self-esteem and self-concept clarity. The interaction of peer pressure and self-esteem was stronger in adolescents with high self-concept clarity (*β* = −0.286, *p* < 0.001) but was not significant in adolescents with low self-concept clarity (*β* = −0.058, *p* = −1.568). The interaction of peer pressure and self-concept clarity was stronger in adolescents with high self-esteem (*β* = −0.289, *p* < 0.001) but was not significant in adolescents with low self-esteem (*β* = −0.062, *p* = 1.509).

**Table 5:** Conditional moderating effects of self-esteem and self-concept clarity.

Participants	Interaction	Effect	SE	*t*	*p*	Bootstrap LLCI	Bootstrap ULCI
Low self-concept clarity	Peer pressure × self-esteem	−0.058	0.037	−1.568	0.117	−0.131	0.015
Moderate self-concept clarity		−0.172^***^	0.039	−4.457	<0.001	−0.248	−0.096
High self-concept clarity		−0.286^***^	0.050	−5.712	<0.001	−0.384	−0.188
Low self-esteem	Peer pressure × self-concept clarity	−0.062	0.041	−1.509	0.132	−0.142	0.019
Moderate self-esteem		−0.176^***^	0.037	−4.810	<0.001	−0.247	−0.104
High self-esteem		−0.289^***^	0.043	−6.660	<0.001	−0.375	−0.204

*N* = 830. Bootstrap sample size = 5,000. SE, standard error; LL, low limit; CI, confidence interval; UL, upper limit. ^*^p < 0.05, ^**^p < 0.01.

Specifically (see Table 6), for adolescents with low self-concept clarity, the effect of peer pressure on mobile social media addiction was significant at low self-esteem (*β* = 0.453, *p* < 0.001) and high levels of self-esteem (*β* = 0.337, *p* < 0.001). For adolescents with high self-concept clarity, the effect of peer pressure on mobile social media addiction was significant at low self-esteem (*β* = 0.329, *p* < 0.001) but was not significant at moderate self-esteem (*β* = 0.044, *p* = 0.429). Peer pressure even negatively predicted mobile social media addiction at high self-esteem for adolescents with high self-concept clarity (*β* = −0.242, *p* < 0.001). Similarly, for adolescents with low self-esteem, the effect of peer pressure on mobile social media addiction was significant at low self-concept clarity (*β* = 0.453, *p* < 0.001) and high self-concept clarity (*β* = 0.32, *p* < 0.001). For adolescents with high self-esteem, the effect of peer pressure on mobile social media addiction was significant at low self-concept clarity (*β* = 0.337, *p* < 0.001) but was not significant at moderate self-concept clarity (*β* = 0.048, *p* = 0.270). Peer pressure even negatively predicted mobile social media addiction at high self-concept clarity for adolescents with high self-esteem (*β* = −0.242, *p* < 0.001).

**Table 6:** Effects of peer pressure on mobile social media addiction at different values of self-esteem and self-concept clarity.

Values of self-concept clarity	Values of self-esteem	Effect	SE	*t*	*p*	Bootstrap LLCI	Bootstrap ULCI
M-SD	M-SD	0.453^***^	0.049	9.211	<0.001	0.356	0.549
	M	0.395^***^	0.047	8.478	<0.001	0.303	0.486
	M + SD	0.337^***^	0.068	4.935	<0.001	0.203	0.471
M	M-SD	0.391^***^	0.061	6.439	<0.001	0.272	0.510
	M	0.219^***^	0.036	6.120	<0.001	0.149	0.290
	M + SD	0.048	0.043	1.104	0.270	−0.037	0.132
M + SD	M-SD	0.329^***^	0.091	3.615	<0.001	0.151	0.508
	M	0.044	0.055	0.792	0.429	−0.065	0.153
	M + SD	−0.242^***^	0.053	−4.554	<0.001	−0.346	−0.138

*N* = 830. Bootstrap sample size = 5,000. SE, standard error; LL, low limit; CI, confidence interval; UL, upper limit. ^*^p < 0.05, ^**^p < 0.01.

Table 7 presents the results of gender differences in the moderating effects of self-esteem and self-concept clarity. The three-way interaction of peer pressure, self-esteem, and gender was insignificant (*β* = 0.075, *p* = 0.062). The three-way interaction of peer pressure, self-concept clarity, and gender was also insignificant (*β* = −0.088, *p* = 0.167). In other ways, there were no significant gender differences in the moderating effects of self-esteem and self-concept clarity.

**Table 7:** Gender differences in the moderating effects of self-esteem and self-concept clarity.

Interaction	Effect	SE	*t*	*p*	Bootstrap LLCI	Bootstrap ULCI
Peer pressure × Self-esteem × Gender	0.075	0.062	1.226	0.220	−0.045	0.196
Peer pressure × Self-concept clarity × Gender	−0.088	0.637	−1.384	0.167	−0.213	0.037

*N* = 830. Bootstrap sample size = 5,000. SE, standard error; LL, low limit; CI, confidence interval; UL, upper limit. ^*^p < 0.05, ^**^p < 0.01.

## 4. Discussion

Social media is increasingly playing a critical role in people’s daily life. Mobile social network services have become an essential core function of mobile phones. However, the following problem of mobile social media addiction occurs and develops into one of the typical problem behaviors among adolescents. This study examined the effect of peer pressure on adolescents’ mobile social media addiction and the moderating effects of self-esteem and self-concept clarity. The results showed that both self-esteem and self-concept clarity significantly alleviated the effect of peer pressure on mobile social media addiction, and there was a significant interaction between the two moderators. The reasons and implications of the results are discussed below.

First, this study found that peer pressure on mobile phone use significantly predicted adolescent mobile phone addiction. Consistent with this result, previous research has shown that peer pressure on Internet use was positively associated with adolescent Internet addiction (7). Mobile phones are increasingly popular among adolescents, and mobile phone use has become the normal behavior of peer groups. Adolescents inevitably face the pressure of maintaining peer relationships using mobile phones in daily life. Peer pressure on mobile phones has become the most important type of peer pressure in the Internet era. According to the peer norm influence theory (10), if adolescents do not comply with peer norms (using mobile phones), they may be snubbed and rejected by peers. However, due to their immature self-control ability (41), many adolescents tend to overuse mobile phones to maintain and develop peer relationships, which will increase the risk of mobile social media addiction. The result of the association between peer pressure and mobile social media addiction and previous results of the association between peer pressure and Internet addiction indicate that peer pressure is a typical risk factor for adolescent behavior addiction in the mobile internet era. Both the general Internet addiction/mobile phone addiction and the specific type of mobile phone addiction are influenced by peer pressure.

Second, this study revealed that self-esteem buffered the association between peer pressure on mobile phone use and social media addiction. The effect of peer pressure on mobile social media addiction was significant only in adolescents with low self-esteem rather than high self-esteem. This result is consistent with sociometer theory (27), which highlights the impact of self-esteem on the social self-evaluation process. This result also coincides with previous research demonstrating the protective effect of self-esteem in buffering negative environmental factors (31, 32). Adolescents with high self-esteem have a positive evaluation of themselves and are less dependent on others’ views and behaviors (46). They are less likely to gain attention and recognition by catering to others’ views and choices. Previous studies have also found that adolescents with high self-esteem are less sensitive to rejection in interpersonal communication (47). Since they are not afraid of peer rejection, they are more resistant to peer pressure on mobile phone use. In addition, prior research has also found that people with low self-esteem have a high fear of missing, and they are more afraid of missing information on social network sites (48, 49), which promotes them to use social network sites very frequently. High self-esteem helps adolescents reduce the fear of missing out (48). Adolescents with high levels of self-esteem will not pay too much attention to the information on social network sites, which will reduce the risk of mobile social media addiction.

Third, this study also demonstrated that self-concept clarity buffered the association between peer pressure on mobile phone use and social media addiction. The effect of peer pressure on mobile social media addiction was significant only in adolescents with low self-concept clarity rather than high self-concept clarity. This result also coincides with previous research indicating the protective effect of self-concept clarity in buffering negative environmental factors (33). Self-concept clarity could regulate the effect of self-evaluation information and encourage people to make decisions and guide behaviors relying on self-information rather than others’ opinions or behaviors (29, 30). Compared to individuals with low self-concept clarity, adolescents with high self-concept clarity have a clearer and firmer self-concept (39). Their views and behaviors are more determined by their internal self-concept and less affected by external pressure. Therefore, they can better adhere to themselves even in the face of peer pressure. Moreover, social network sites are full of all kinds of information. There are contradictions and conflicts between diversified information (50), which may produce a stronger information load for individuals with high self-concept clarity. Research has found that information load is a key factor in promoting individuals to stop using social network sites (51). It may also help reduce the risk of mobile social media addiction among adolescents with high self-concept clarity.

In addition, the present study found no gender differences in the moderating roles of self-esteem and self-concept clarity, but the two moderators (i.e., self-esteem and self-concept clarity) significantly interact. The moderating role of self-esteem was more potent in adolescents with high self-concept clarity than those with low self-concept clarity. Similarly, the moderating role of self-concept clarity was more potent in adolescents with high self-esteem than in those with low self-esteem. In other words, the two protective factors can mutually enhance. Previous studies have found that the negative impact of risk factors on self-evaluation is stronger in individuals with lower self-concept clarity (33). The result indicates that as two core indicators of self-concept, self-esteem and self-concept clarity, can exert a stronger effect when the two factors act together than when they act alone. Therefore, educational intervention on self-concept should consider these two components simultaneously.

Some limitations should be noted. First, the causal relationship between peer pressure and mobile social media addiction cannot be strictly confirmed by the cross-sectional design we used. Future research should consider using a longitudinal design or experimental study. Second, this study only focused on social media addiction without including different types of mobile phone addiction. In addition to mobile social media addiction, peer pressure may cause other types of mobile phone addiction, such as mobile game addiction. Future research should consider testing whether peer pressure exerts different effects on different mobile phone addictions and whether the moderating effects of self-esteem and self-concept clarity will show differences in different types of mobile phone addiction. Third, academic performance is an important factor in adolescence, but we did not include it in the analysis. Academic performance was closely associated with self-esteem and mobile phone addiction (17, 52). Future research may explore whether academic performance played an important role in the association between peer pressure, self-esteem, and mobile phone addiction.

Despite the above limitations, our findings still bring about important implications. First, this is one of the first studies documenting the impact of peer pressure on mobile social media addiction. The findings expand previous research on the association between peer pressure and Internet addiction. Second, this study further uncovers the critical roles of self-esteem and self-concept clarity in buffering the impact of peer pressure. These results contribute to understanding how to buffer the effect of peer pressure and how to reduce mobile social media addiction. Third, this study has specific implications for preventing and intervening in mobile social media addiction. Since peer pressure directly increases the risk for mobile social media addiction, parents and educators should help adolescents understand the harm of peer pressure, stay away from deviant peers, and avoid the impact of negative peer pressure. Besides, since self-esteem and self-concept clarity can effectively buffer the influence of peer pressure, parents and educators should help adolescents develop positive self-evaluations and clear self-concepts.

In conclusion, we conducted a moderation analysis to analyze the moderating roles of self-esteem and self-concept clarity in the association between peer pressure on mobile phone use and mobile social media addiction. Peer pressure directly predicted adolescent mobile social media addiction. Both self-esteem and self-concept clarity effectively moderated the effect of peer pressure. Peer pressure had a significant impact on mobile social media addiction only in adolescents with low self-esteem or self-concept clarity. In addition, there was a significant interaction between the two moderators. The moderating role of self-esteem was more potent in adolescents with high self-concept clarity but not significant in low self-concept clarity adolescents. The moderating role of self-concept clarity was more potent in adolescents with high self-esteem but not significant in low self-esteem adolescents. The findings highlight the protective roles of self-esteem and self-concept clarity in the mobile Internet era. Enhancing self-esteem and self-concept clarity will be an effective way to protect adolescents who experience high peer pressure from mobile social media addiction.

## Data availability statement

The raw data supporting the conclusions of this article will be made available by the authors, without undue reservation.

## Ethics statement

The studies involving human participants were reviewed and approved by the Ethics Committee of Guangzhou University. Written informed consent to participate in this study was provided by the participants’ legal guardian/next of kin.

## Author contributions

XX contributed to the conceptualization, methodology, investigation, and writing—original draft, review, and editing. WH contributed to the resources, and writing—review and editing. QL contributed to the conceptualization, methodology, investigation, formal analysis, and writing—original draft, review, and editing. All authors contributed to the article and approved the submitted version.

## Funding

This work was supported by the Programs of the National Social Science Fund of China (No. 21BDJ054 and No.18CRK009) and the Program of the Fund of Philosophy and Social Science of Guangdong Province (No. GD20CXL05).

## Conflict of interest

The authors declare that the research was conducted in the absence of any commercial or financial relationships that could be construed as a potential conflict of interest.

## Publisher’s note

All claims expressed in this article are solely those of the authors and do not necessarily represent those of their affiliated organizations, or those of the publisher, the editors and the reviewers. Any product that may be evaluated in this article, or claim that may be made by its manufacturer, is not guaranteed or endorsed by the publisher.
